# Cognitive Reserve in *Granulin*-Related Frontotemporal Dementia: from Preclinical to Clinical Stages

**DOI:** 10.1371/journal.pone.0074762

**Published:** 2013-09-09

**Authors:** Enrico Premi, Stefano Gazzina, Marco Bozzali, Silvana Archetti, Antonella Alberici, Mara Cercignani, Angelo Bianchetti, Roberto Gasparotti, Marinella Turla, Carlo Caltagirone, Alessandro Padovani, Barbara Borroni

**Affiliations:** 1 Centre for Neurodegenerative Disorders, University of Brescia, Brescia, Italy; 2 Neuroimaging Laboratory, Santa Lucia Foundation IRCCS, Rome, Italy; 3 III Laboratory of Analysis, Brescia Hospital, Brescia, Italy; 4 Brighton and Sussex Medical School, Clinical Imaging Centre, University of Sussex, Brighton, United Kingdom; 5 Geriatric Research Group, Brescia, Italy; 6 Neuroradiology Unit, University of Brescia, Brescia, Italy; 7 Neurology Unit, ValleCamonica Hospital, Brescia, Italy; 8 Department of Neuroscience, University of Rome “Tor Vergata”, Rome, Italy; University G. D'Annunzio, Italy

## Abstract

**Objective:**

Consistent with the cognitive reserve hypothesis, higher education and occupation attainments may help persons with neurodegenerative dementias to better withstand neuropathology before developing cognitive impairment. We tested here the cognitive reserve hypothesis in patients with frontotemporal dementia (FTD), with or without pathogenetic *granulin* mutations (*GRN+* and *GRN-*), and in presymptomatic *GRN* mutation carriers (*aGRN+*)*.*

**Methods:**

Education and occupation attainments were assessed and combined to define Reserve Index (RI) in 32 FTD patients, i.e. 12 *GRN+* and 20 *GRN*-, and in 17 *aGRN+*. Changes in functional connectivity were estimated by resting state fMRI, focusing on the salience network (SN), executive network (EN) and bilateral frontoparietal networks (FPNs). Cognitive status was measured by FTD-modified Clinical Dementia Rating Scale.

**Results:**

In FTD patients higher level of premorbid cognitive reserve was associated with reduced connectivity within the SN and the EN. EN was more involved in FTD patients without *GRN* mutations, while SN was more affected in *GRN* pathology. In *aGRN+*, cognitive reserve was associated with reduced SN.

**Conclusions:**

This study suggests that cognitive reserve modulates functional connectivity in patients with FTD, even in monogenic disease. In *GRN* inherited FTD, cognitive reserve mechanisms operate even in presymptomatic to clinical stages.

## Introduction

The cognitive reserve hypothesis posits that lifetime intellectual enrichment lessens the negative impact of neurological diseases on the cognitive status [1]. When the neurocognitive processing is challenged by brain disease, individuals with greater premorbid cerebral efficiency are able to withstand better the neurocognitive challenges, thus showing a relative resilience to cognitive impairment [1]. To account for these clinical observations, the concepts of cognitive and brain reserves have been developed [2], with the hypothesis that phenomena of brain plasticity might represent the underlying neurobiological substrate. It has been recently demonstrated that Alzheimer’s disease (AD) patients with higher levels of formal education (a proxy measure of cognitive reserve) require more brain atrophy in those regions typically targeted by the pathology to exhibit the same level of cognitive decline shown by AD patients with lower education levels [3].

In the same view, the role of cognitive reserve hypothesis has been investigated also in Frontotemporal Dementia (FTD), a neurodegenerative disease characterized by behavioral disorders, language impairment, and deficits of executive functions as most typical clinical features [4,5]. Literature data suggested that education and occupational attainments might act as a proxy measure of reserve capacity in FTD, as well as AD [6]. Furthermore, as in AD [7], cognitive reserve in FTD is still in action even in the presence of an unfavorable genetic background [8].

FTD has a strong genetic background, and a number of genes causative of autosomal dominant forms have been identified so far. Among others, *Granulin* (*GRN*) mutations, inducing a loss of 50% functional Progranulin [9,10], are present in a proportion of patients whose most typical clinical presentations include the behavioral variant of Frontotemporal Dementia (bvFTD) and the agrammatic variant of Primary Progressive Aphasia (avPPA). *GRN* mutations are, by definition, inherited at birth, with the disease onset that typically occurs at the 5^th^-6^th^ decade of life, although there are rare subjects who carry pathogenetic variation in their late life, without any sign of the disease. This means that FTD patients carrying *GRN* mutation have a completely normal life until their fifties, and if the disease begins, *GRN* mutation carriers have a worse clinical prognosis than FTD patients without mutations [11]. However, a small quote of mutation carriers show an incomplete penetrance, thus suggesting the possibility of genetic or environmental disease modifiers.

As many cases of FTD are inherited, the role of cognitive reserve in patients with monogenic disease, i.e. *GRN*-disease, still needs to be established moving from preclinical to symptomatic stages.

Imaging genetics is a growing field that is shedding light for new discoveries in neuroscience [12]. Magnetic resonance imaging (MRI) has become an increasingly powerful tool for human brain investigation, and using different modalities, has been successfully used to investigate different pathophysiological aspects of the brain tissue in the presence of neurodegeneration [13,14]. Beyond structural MRI, resting state functional MRI (fMRI) has shown the ability to provide measures of functional brain connectivity, based on the evidence that different brain regions are functionally synchronized at rest, and connected regions are supposed to define common networks subserving complex brain functions. In the presence of neurodegeneration, the loss of functional brain connectivity is likely to account for cognitive disabilities and even for some gray matter loss secondary to neuronal disconnection [15]. From resting state fMRI data (i.e., fMRI time series collected while subjects lie vigilant but at rest in the scanner), several networks can be extracted in a data-drive fashion, by using the so-called Independent Component Analysis algorithm [16]. Initial resting-state studies in FTD described a divergent relationship between Default Mode Network (DMN) and Salience Network (SN) connectivity, with attenuated connectivity of SN [17,18], whose activity is related to the autonomic/interior processing and the "salience" of the stimulus, like the emphatic mechanisms and the emotional aspect of pain [19,20]. However, recently other networks have been described as involved in FTD, in particular the Executive Network (EN), and Frontoparietal Networks (FPNs) [21]. The areas belonging to EN have been hypothesized to provide bias signals to other areas of the brain in order to improve cognitive control [22]. Furthermore, the cortical regions sustaining EN are specifically involved in Frontotemporal Dementia, playing a role in the disease progression [23]. On the other side, FPNs have been related to top-down modulation of attention and working memory [24]. From previous studies FPNs seem to be involved in the selection of relevant environmental information, which could be important for the integration between environmental sensory stimulus and behavioral goals and expectations [25]. Furthermore, in FTD these changes are more pronounced in patients with *GRN* mutations; at the moment, only two studies have explored functional network connectivity alterations in presymptomatic *GRN* carriers showing impaired resting state functional connectivity in the network primarily involved in the pathology (i.e. SN) [18,26]. Resting state fMRI may contribute to clarify the interaction between genetic and environmental factors in modulating the occurrence of clinical symptoms and to define a theoretical model of disease progression, moving from the presymptomatic stage to clinical presentation.

With these caveats in mind, the current study uses resting state fMRI to investigate the relationship between lifetime intellectual enrichment and patterns of brain connectivity in patients with FTD, with and without *GRN* pathogenetic mutations, and in presymptomatic *GRN* mutation carriers.

## Methods

### Subjects

Subjects entering the present study were partly the same as those recruited for a previous investigation [18] (N= 30), and in part (N=19) newly recruited. In the former case, subjects were invited to attend again the Centre for Ageing Brain and Neurodegenerative Disorders, at University of Brescia (Brescia, Italy), to collect data for the assessment of cognitive reserve. In the latter case, subjects were also asked to undergo the MRI protocol, as detailed below. The studied sample included 32 patients with FTD all genetically characterized for the presence/absence of *GRN* and *MAPT* mutations and C9orf72 hesanucleotide expansion. Twelve of them proved to be carriers of *GRN Thr272fs* mutation (*GRN+*), while the remaining 20 proved to be non-carriers of screened genetic variations (*GRN-*). The current study included also 17 asymptomatic carriers of *GRN Thr272fs* mutation (*aGRN+*; all siblings of GRN+ FTD patients). Nine of them had already taken part in our previous study [18], while the remaining 8 were newly recruited.

All FTD patients met current clinical diagnostic criteria for bvFTD [27] (18 cases) or avPPA [28] (14 cases). To increase as much as possible the confidence of a correct diagnosis of FTD in patients without *GRN Thr272fs* mutation, they had to be clinically and neuropsychologically followed-up for at least 2 years, at the time of recruitment.

All patients underwent a clinical and neurological evaluation, a routine laboratory examination, and conventional brain MRI before entering this study, to rule out any potential alternative diagnosis. An extensive neuropsychological assessment in both patients and asymptomatic siblings, including the FTD-modified Clinical dementia Rating scale (FTD-modified CDR), was administered, as previously described [18].

Written informed consent (from the subject or from the responsible guardian if the subject was incapable) was obtained, for each procedure, before study initiation, including blood collection from venous puncture, genetic analysis, and MRI scanning. The research protocol was approved by the ethics committee of the Hospital (Comitato Etico, Azienda Ospedaliera “Spedali Civili”, Brescia, Italy). The work conformed to the Helsinki Declaration.

This research received no specific grant from any funding agency in the public, commercial or not-for-profit sectors.

Authors have no competing interests, or other interests that might be perceived to influence the results and/or discussion reported in this article.

### Assessment of Cognitive reserve

Cognitive reserve was assessed using education and occupational attainment as proxy measures. Education was defined as the number of completed years of formal education, including university or apprenticeship (only in the case a formal educational program was associated). Occupational attainment was defined as previously described [6], with a score ranging from 0 to 4, corresponding to the last employment of each subject. Considering the distribution of occupational score in our sample (ranging from 1 to 3, with no patients with scores 0 or 4), we transformed the educational level (continuous variable) in a three-level categorical variable (1=0-5 years, 2=6-9 years, 3=>9 years). These two categorical variables were summed up to obtain a global Reserve Index (RI), in order to evaluate the combined effect of these variables.

### Granulin sequencing

Genomic DNA was extracted from peripheral blood using a standard procedure. All the 12 exons plus exon 0 of *GRN*, and at least 30 base pairs (bp) of their flanking introns were evaluated by polymerase chain reaction (PCR) and subsequent sequencing. *GRN Thr272fs* (*g*.1977_*1980 delCACT*) was tested as previously described [29].

### Statistics for demographic, laboratory, and cognitive reserve variables

SPSS package (v. 17.0, Chicago, IL, USA) was used to run statistics for group differences in demographic and clinical characteristics, laboratory measures, and cognitive reserve. Group comparisons were assessed by Mann-Whitney test or χ^2^ test, setting the statistical threshold to *P* values Bonferroni’s corrected ≤ 0.05.

### MRI acquisition

All imaging was obtained using a 1.5 T magnetic resonance scanner (Siemens Symphony, Erlangen, Germany), equipped with a circularly polarized transmit-receive coil, as previously published [18]. Resting state fMRI data were preprocessed using Statistical Parametric Mapping (SPM8) (www.fil.ion.ucl.ac.uk/spm/) for image preprocessing and statistical comparison, and the Group independent component analysis (ICA) for fMRI toolbox (GIFT, icatb.sourceforge.net/) for network identification. For each subject the first 4 volumes of the fMRI series were discarded to allow for T1 equilibration effects. The preprocessing steps included correction for head motion, compensation for slice-dependent time shifts, normalization to the EPI template in Montreal Neurological Institute coordinates provided with SPM8, and smoothing with a 3D Gaussian Kernel with 8 mm^3^ FWHM. Then, all images were filtered by a phase-insensitive bandpass filter (pass band 0.01–0.08 Hz) to reduce the effect of low frequency drift and high frequency physiological noise.

Briefly, group ICA for fMRI toolbox first concatenates the individual data across time, and then produces a computation of subject specific components and time courses. For all subjects grouped together, the toolbox performed the analysis in 3 steps: (1) data reduction, (2) application of the FastICA algorithm, and (3) back-reconstruction for each individual subject [30]. ICA analysis was employed to identify 40 independent components, using the Minimum Description Length Criterion for the dimension determination [31]. Statistical reliability of independent component decomposition was evaluated using the ICASSO Toolbox, implemented in GIFT [32] running FastICA algorithm 10 times with different initial conditions and bootstrapped data sets. Results were converted to *Z*-scores. The 40 components were reviewed, and compared, by computing the spatial correlation coefficient, to customized templates of the networks affected by the pathology, according to literature data [21] i.e. dorsal and ventral Salience Network (SN), Default Mode Network (DMN), Executive Network (EN), Frontoparietal Networks (FPNs) and Dorsal Attention Network (AN) [21]. This procedure was performed using the tool for spatial sorting of the components available with GIFT. Every subject’s *Z*-score maps corresponding to these resting state networks were used for cross-subject analyses. For the purpose of the present study, subjects were divided into 3 separate groups: patients with FTD *GRN Thr272fs* mutation carriers (*GRN+, n* = 12); patients with FTD non mutation carriers (*GRN-, n* = 20); asymptomatic subjects FTLD *GRN Thr272fs* mutation carriers (*aGRN+, n* = 17). Age, gender, dementia severity scored with FTD-CDR scale and total grey matter volume were entered as covariates of no interest.

For each considered network, contrasts were designed to assess the correlation of RI with functional connectivity in FTD patients (either in FTD-GRN*+* and FTD-GRN*-*); at this purpose, a linear regression analysis between RI (as independent variable) and network resting-state functional connectivity (dependent variable) was performed in each group (FTD-GRN*+* and FTD-GRN*-*); then, a difference of slope (interaction analysis) was used to evaluate different reserve effects in *GRN+* and *GRN-*; in particular, the statistical differences between the regression of RI scores in FTD-GRN*+* and FTD-GRN- groups were studied (FTD-GRN*+ <* FTD-GRN*-*; FTD-GRN*- <* FTD-GRN*+*). [33]. In the *aGRN+* group a linear regression analysis using RI scores was performed to study the reserve effect. *P*-values were defined at p<0.001 uncorrected, and only clusters surviving at FWE<0.05 were considered. Threshold was set at 30 voxels.

## Results

### Subjects

As shown in Table **1**, there was a significant difference in age at evaluation (*P*=0.024) between FTD *GRN+* and *GRN-*. No significant differences in disease duration, gender, and clinical phenotypes distribution between *GRN+* and *GRN*- were found. As expected, patients with *GRN+* had a higher rate of positive family history for dementia (91.7%) than those with *GRN*- (40%, *P*=0.008).

**Table 1:** Clinical and demographic characteristics of the subjects studied.

**Variable**	**FTD (all)**	***GRN+***	***GRN-***	***aGRN+***
	n=32	n=12	n=20	n=17
Age at evaluation^, y	64.7±6.8	61.3±5.2	67.4±7.2	40.3±9.7
Age at onset, y	62.2±6.9	59.2±6.4	64.0±6.3	-
Disease duration, y	2.5±2.3	2.1±2.0	3.4±2.7	-
Gender, female % (n)	43.8 (14)	66.7 (8)	25.0 (5)	41.2 (7)
Family history*, positive % (n)	59.4 (19)	91.7 (11)	40.0 (8)	-
Clinical phenotype, bvFTD %(n)	56.3(18)	50.0 (6)	60.0 (12)	-
FTD-CDR^	5.3±3.3	6.1±3.6	4.5±3.1	-
***CognitiveReserveIndex***				
Reserve Index**	3.65±1.32	3.00±0.85	3.40±1.27	4.41±1.33

FTD: Frontotemporal dementia; GRN+: FTD patients carrying Granulin Thr272fs mutation; GRN-: FTD patients without Granulin Thr272fs mutation; aGRN+: asymptomatic subjects carrying Granulin Thr272fs mutation; FTD-CDR: Frontotemporal dementia modified Clinical dementia rating scale.

GRN+ vs. GRN-, ^ P=0.024; *P=0.008. t-test, otherwise specified or Chi-square test were performed, as required. Results are expressed as mean ± standard deviation. Number of subjects between brackets. One way-ANOVA between the three groups (*GRN+, GRN-, aGRN+*), **P=0.007. See text for further details.

*GRN+* and *GRN*- showed comparable levels of education and occupation levels. Furthermore, *aGRN+* presented a statistically significant difference in reserve index score (*P*=0.007) than the other two groups.

### Resting-State fMRI analysis in FTD patients

**Figure 1:**
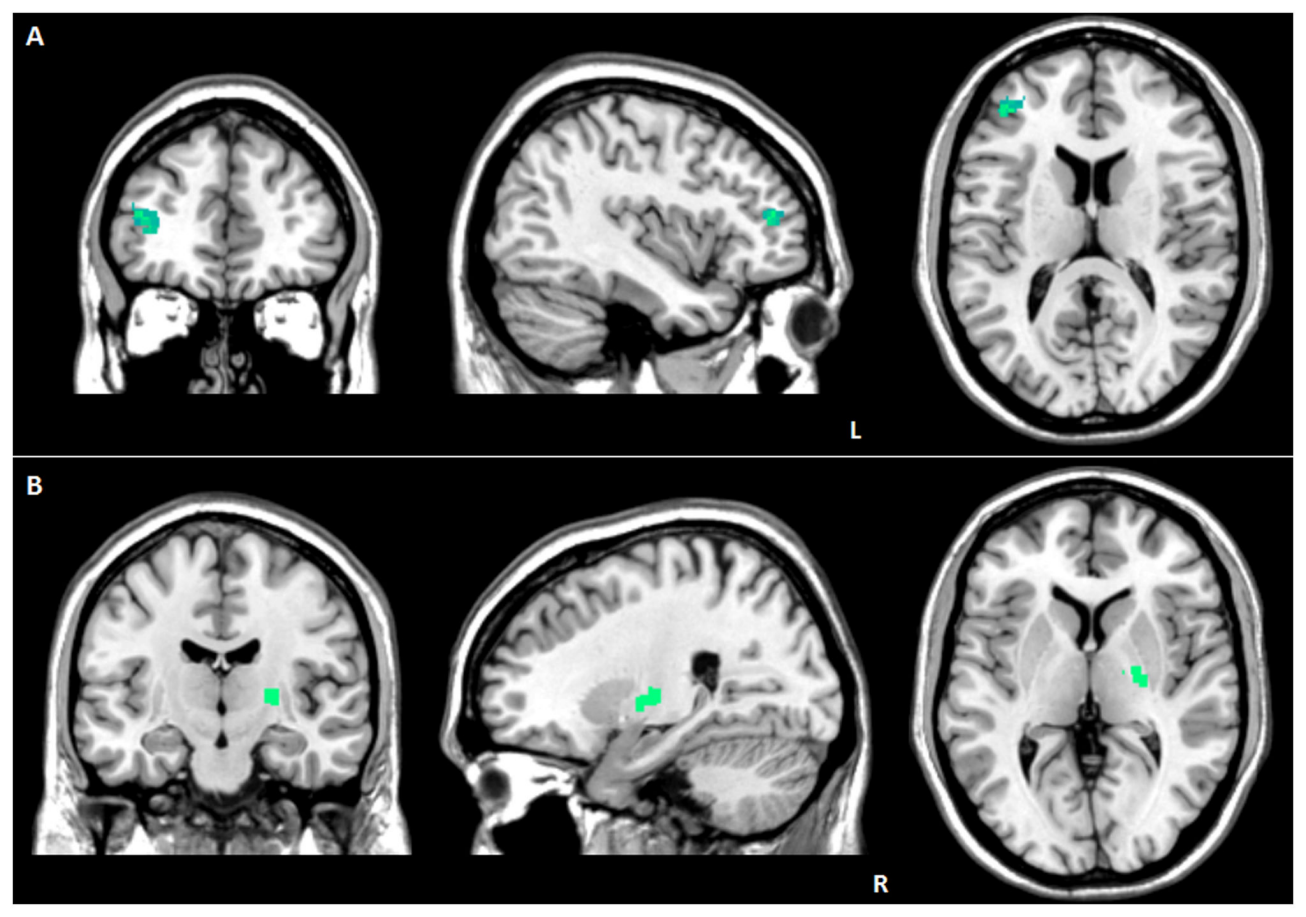
Correlation between Reserve Index and functional connectivity in FTD patients. (A) reduced EN connectivity in the left middle frontal gyrus; (B) reduced ventral SN connectivity in the right lentiform nucleus. L: left. R: right. Statistical threshold: P values cluster level FWE corrected < 0.05. See text for further details.

In FTD (considering both groups, FTD-GRN*+* and FTD-GRN*-*) EN showed reduced connectivity in the left middle frontal gyrus (83 voxels; -40, 44, 14; P FWE-cluster level=0.01; T=5.16) for increasing values of RI (Figure **1A**). The same relationship was observed when considering ventral SN, in the right lentiform nucleus (76 voxels; 24, -14, 4; P FWE-cluster level=0.01; T=5.49) (Figure **1B**).

No significant correlations between RI and dorsal SN, DMN, dorsal attention and FPNs connectivity were evident.

**Figure 2:**
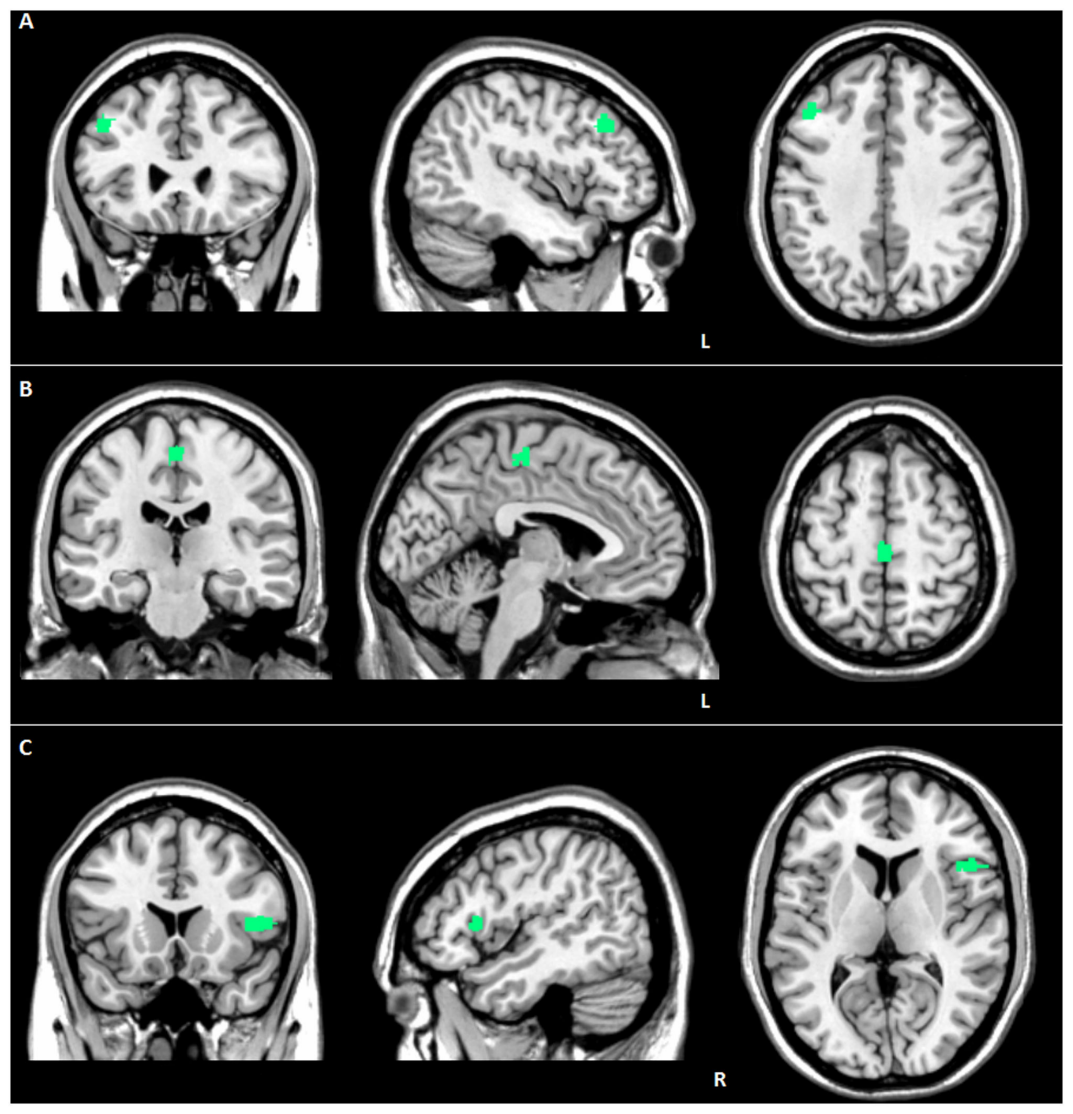
Difference of slope analysis between FTD-GRN*+* and FTD-GRN- patients. (A) reduced EN connectivity in the left middle frontal gyrus in FTD-GRN- patients, as compared to FTD-GRN*+*; (B) reduced ventral SN connectivity in the left medial frontal gyrus in GRN+ patients, as compared to FTD-GRN *-*; (C) reduced dorsal SN connectivity in the right precentral gyrus in FTD-GRN*+* patients, as compared to FTD-GRN *-*. L: left. Statistical threshold: P values cluster level FWE corrected < 0.05. See text for further details.

When applying slope analysis, in *GRN*- a lower functional connectivity in the EN in the same region (69 voxels; -44, 26, 38; P FWE-cluster level=0.03; T=6.69) (Figure **2A**) was observed, compared to *GRN+*. By the same analysis, in *GRN+* a greater damage in both ventral (left medial frontal gyrus; 84 voxels; -4, -22, 58; P FWE-cluster level=0.007; T=6.07) (Figure **2B**) and dorsal (right precentral gyrus; 77 voxels; 48, 18, 8; P FWE-cluster level=0.02; T=5.93) (Figure **2C**) SN emerged, compared to *GRN-*. 

### Resting-State fMRI analysis in *aGRN+* subjects

**Figure 3:**
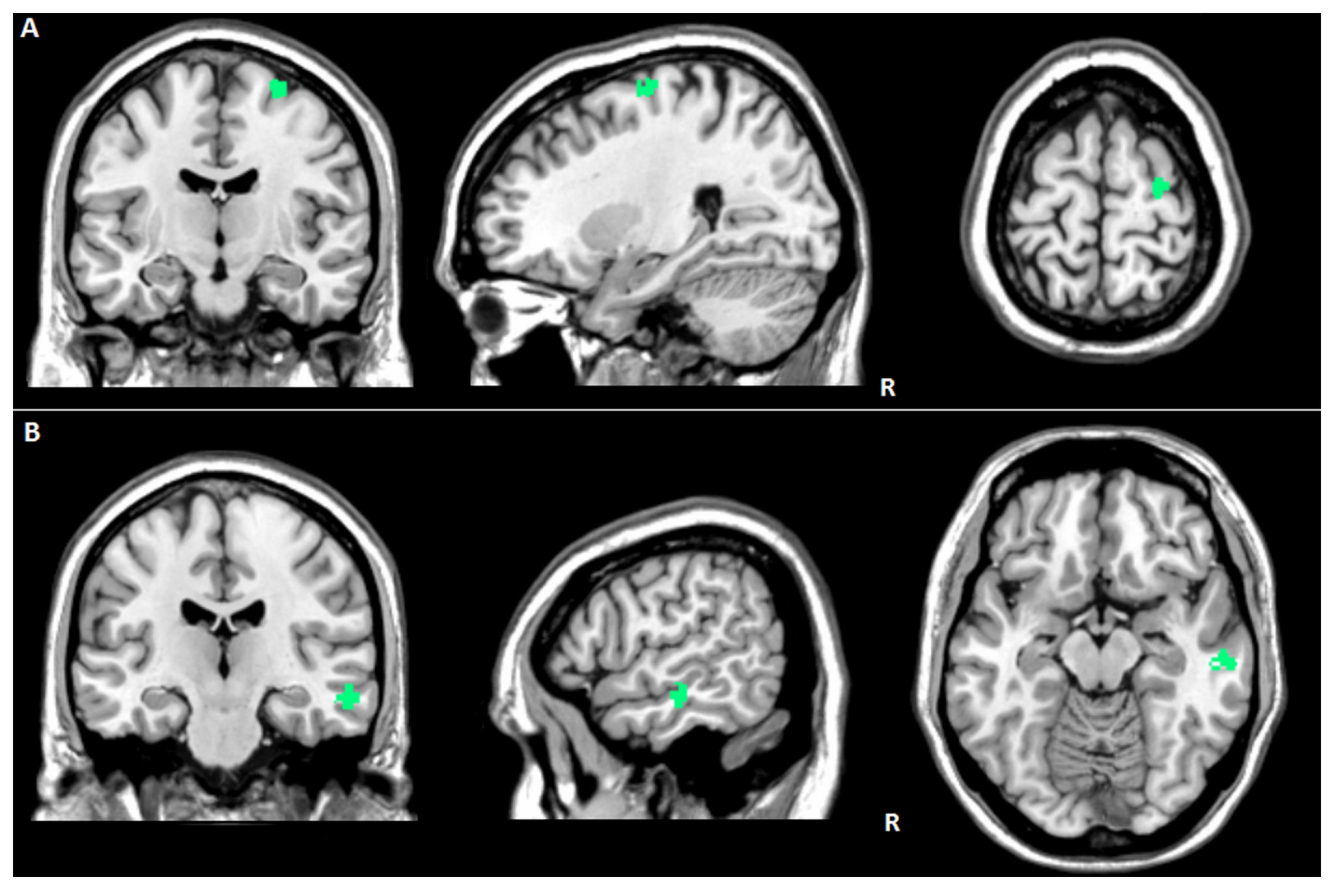
Correlation between Reserve Index and functional connectivity in presymptomatic *GRN* carriers (*aGRN+*). (A) Reduced ventral SN connectivity in the right precentral gyrus; (B) Reduced dorsal SN connectivity in the right middle temporal gyrus. L: left. R: right. Statistical threshold: P values cluster level FWE corrected < 0.05. See text for further details.

In *aGRN+*, RI was inversely related to functional activation of the ventral SN in the right precentral gyrus (47 voxels; 26, -12, 68; P FWE-cluster level=0.02; T=7.92) (Figure **3A**) and of the dorsal SN in the right middle temporal gyrus (59 voxels; 58, -20, -16; P FWE-cluster level=0.01; T=5.92) (Figure **3B**). No relationships between RI and functional connectivity in EN, DMN, dorsal attention and FPNs emerged.

## Discussion

In this study we used resting state fMRI to investigate the relationship between lifetime intellectual enrichment and patterns of brain functional connectivity in FTD and in presymptomatic disease stages. We considered patients with and without pathogenetic *GRN* mutations, to assess the role of cognitive reserve in monogenic inherited disease, and asymptomatic subjects carrying *GRN* mutations, to evaluate how cognitive reserve acts on functional neuronal networks almost twenty years before the disease onset.

When considering the whole FTD group, the main finding was that education and occupation, taken together as Reserve Index, modulate functional connectivity in those networks mainly affected by FTD. In addition, slope analysis revealed that there may be a different network involvement in *GRN*-driven pathology compared to sporadic disease, with predominant SN changes in *GRN* patients.

We interpreted these data supporting the idea that patients with high levels of cognitive reserve successfully compensate with FTD, and need more advanced pathology before they exhibit clinical symptoms, so that for a given degree of dementia severity, high cognitive reserve patients have more pathology. These findings are overall consistent with a large body of previous literature in AD [3,33,34], and more recently in FTD [6,35] in which has been demonstrated that lifetime enrichment was inversely associated with damage in frontotemporal regions, typically involved in the disease [36]. However, to the best of our knowledge, this is the first study investigating the impact of cognitive reserve on functional brain connectivity in neurodegenerative dementias.

In the present study, functional brain connectivity might in principle account not only for the impact of cognitive reserve on brain damage severity. On this subject, only a few fMRI studies in normal ageing [37,38] and in multiple sclerosis [39] have been published, all consistently showing that higher levels of cognitive reserve were associated with reduced task-related activation in typically involved regions. We might therefore speculate that, in our cohort of patients, those individuals with higher cognitive reserve need more disconnecting damage within the networks more remarkably targeted in FTD pathology to exhibit similar cognitive disability, as compared to those with lower cognitive reserve. Furthermore, the presence of *GRN* mutation correlates with the predominant involvement of SN (dorsal and ventral) suggesting a specific role of this network in reserve mechanisms in *GRN-*related FTD patients [8].

The second part of the study was devoted to the assessment of the role of lifestyle enrichment in subjects carrying inherited pathogenetic mutations within *GRN* gene. At the moment no other work has studied the role of cognitive reserve in asymptomatic carriers of *GRN* mutation. Our group has previously demonstrated that presymptomatic carriers show impaired functional connectivity, even in absence of any detectable cognitive or behavioral deficits [18]; a more recent work [26] (that considered either *GRN* and *MAPT* asymptomatic carriers) demonstrated an altered resting state functional connectivity in SN. In line with this findings, our work showed the presence of reserve mechanisms involving both dorsal and ventral SN. Thus, resting state MRI studies, could potentially detect the effect of proxies of reserve antedating structural brain damage.

Our results suggest that compensatory mechanisms are in action almost 20 years before disease onset in those networks typically affected by the pathology, and that these mechanisms involve different areas moving from preclinical to symptomatic stage, probably due to progressive depletion of scaffolding properties [40]. In addition, results show a different pattern of reserve in *GRN* patients, as compared to sporadic FTD patients, and this may account for a different spreading of pathology.

Taken together, it may be supposed that cognitively stimulating lifestyles result in greater elaboration of synaptic networks within the brain in FTD. Concerning monogenic *GRN* disease, life experiences make a unique contribution to cognitive reserve over-and-above genetic disadvantage in preclinical stages of the disease, and become less efficient when disease is overt. Furthermore, recent data on brain cognitive reserve mechanisms supported their intrinsic dynamicity, their evolution during lifetime and the complex influence of lifestyle [40].

We acknowledge some limitations of the present study. Firstly, engagement of cognitive leisure activities such as reading, writing, and other hobbies were not considered here, but they are also likely to contribute in determining the cognitive reserve. The degree and duration of cognitively stimulating variables were not taken into account. Furthermore, we included only *GRN Thr272fs* mutations to avoid confounds, but the effect of other pathogenetic *GRN* variants as well as other mutations leading to FTD, i.e. *MAPT* or *C9orf72*, should be further tested. Finally, longitudinal studies evaluating the effect of cognitive reserve on disease progression and disease onset are warranted.

The current study has utilized fMRI to demonstrate that intellectual enrichment was associated with cerebral efficiency in this disorder for which no disease-modifying treatment is currently available. For this reason, the notion that lifestyle choices can have a direct impact on the brain resilience to FTD pathology opens new perspectives in term of symptom prevention and delaying. This is particularly relevant for individuals carrying pathogenetic mutations who will certainly develop FTD at some point in life. In this sense, future research should investigate whether cognitive interventions, i.e. cognitive strategy training, might improve cognitive efficiency, i.e. cognitive reserve, in patients at early disease stages and in subjects at risk of developing disease, thereby delaying the clinical onset and the progression of neurodegenerative diseases.
